# Effectiveness of tramadol-including multimodal analgesia in spinal surgery: a single-center, retrospective cohort study

**DOI:** 10.1186/s40780-024-00381-7

**Published:** 2024-09-19

**Authors:** Misa Okizuka, Ryo Inose, Satoshi Makio, Yuichi Muraki

**Affiliations:** 1 Department of Pharmacy, Rakuwakai Marutamachi Hospital, 9-7 Matsushita-Cho, Kyoto, Jurakumawari, Nakagyo-ku, Kyoto-shi 604-8401 Japan; 2 Laboratory of Clinical Pharmacoepidemiology, Kyoto Pharmaceutical University, 5 Misasagi Nakauchi-Cho, Kyoto, Yamashina-Ku, Kyoto-Shi 607-8414 Japan; 3 Department of Orthopaedics, Rakuwakai Marutamachi Hospital, 9-7 Matsushita-Cho, Kyoto, Jurakumawari, Nakagyo-ku, Kyoto-shi 604-8401 Japan

**Keywords:** Multimodal Analgesia, Tramadol, Spinal Surgery, Length of stay, Numerical rating scale

## Abstract

**Background:**

Multimodal analgesia (MMA) is recommended for postoperative pain management; however, studies evaluating the effect of tramadol-including MMA on numerical rating scale (NRS)-based postoperative pain levels and the length of stay (LOS) in the hospital are limited. Therefore, this study aimed to compare the before and after effects of tramadol-including MMA application, and assess its effect on postoperative NRS scores and LOS.

**Methods:**

Patients who underwent spinal surgery under general anesthesia at the Rakuwakai Marutamachi Hospital in fiscal years 2020 and 2022 were included in this study. The outcomes between the pre- and post-intervention groups were compared through propensity score matching.

**Results:**

Following propensity score matching, 249 patients were included in each group. MMA application significantly decreased the median LOS from 10 to 9 days (*p* < 0.001). Additionally, the median NRS scores exhibited a significant decrease from 4 to 3 on postoperative day (POD) 3 (*p* = 0.0109) and from 3 to 2 on POD 5 (*p* = 0.0087). Following MMA application, the number of patients receiving additional analgesics decreased significantly, from 38 to 6 (*p* < 0.001).

**Conclusions:**

The introduction of tramadol-including MMA can effectively reduce postoperative pain and decrease the LOS for patients undergoing spinal surgery.

## Background

Postoperative pain and its poor management can adversely affect various organs and the immune system [1], resulting in chronic pain and negatively affecting daily activities [2]. Various factors, including nociceptive, neuropathic, and inflammatory factors, can induce postoperative pain [2]. Multimodal analgesia (MMA), which combines analgesics with different mechanisms of action, has been recommended for managing diverse types of pain [3].

Furthermore, enhanced recovery after surgery (ERAS) protocols, including systematic methods to facilitate early recovery before, during, and after surgery, recommend MMA to improve surgical outcomes and decrease the length of stay (LOS) in the hospital [4, 5]. The ERAS principles were initially established for colorectal surgery and have been extended to orthopedic surgery [5].

Recently, MMA has been reported to be effective in spinal surgery, regarding decreased opioid use [6, 7] and improved pain scores [6]. However, extensive investigation on the use of MMA in spinal surgery is lacking. Although non-opioids such as gabapentinoids, acetaminophen, ketamine, non-steroidal anti-inflammatory drugs (NSAIDs), and local anesthetics have shown efficacy in MMA [8], optimal drug combinations have not been established. Tramadol is a weak opioid drug and suppresses nociceptive and neuropathic pain through its combined effects of noradrenaline and serotonin reuptake inhibition [9]. Tramadol may be used for alleviating postoperative pain after spinal surgery, as surgery may induce neuropathic pain in addition to acute pain. However, there are limited studies that have evaluated the effects of tramadol-including MMA on postoperative pain levels using the numerical rating scale (NRS) scores and LOS in the hospital. Therefore, this study aimed to compare the effects of tramadol-including MMA before and after its introduction and assess its effects on postoperative NRS scores and LOS.

## Methods

### Study design and subjects

This retrospective study included patients who underwent spinal surgery under general anesthesia at a private hospital in Kyoto, Japan, centered on orthopedic care during fiscal year (FY)2020 and FY2022. According to the FY, the patients were categorized into the pre-intervention group (FY2020) and the post-intervention group (FY2022). The exclusion criteria were as follows: patients who were not prescribed pre-agreed analgesics and those for whom access to medical records was not possible.

### Tramadol-including MMA application

Before introducing MMA, patients received a single dose of celecoxib (800 mg) after dinner on the day of surgery. From postoperative day (POD) 1, the patients were administered celecoxib (400 mg) twice daily after meals. After POD 1, the patients were administered a single dose of celecoxib (400 mg) twice daily in the morning and evening after meals. After introducing MMA, celecoxib administration was continued in the patients, following the previous regimen. Additionally, the patients were administered 1,000 mg of acetaminophen thrice daily from breakfast on POD 1. From dinner onward on POD 2, 25 mg of tramadol thrice daily after each meal was added to the regimen. The dose of tramadol reported in this study was lower than the dose reported in a prior study [10]. This adjustment was made with safety in mind and in consultation with the physician. Celecoxib was not administered to patients with creatinine clearance (Ccr) of < 40 mL/min, both before and after MMA application. Patients with a Ccr of < 40 mL/min before MMA application were administered acetaminophen instead of celecoxib as an abortive dose. Each analgesic was discontinued or reduced after discussion among the physician, pharmacist, and patient once pain control was satisfactory while taking it. Narcotic analgesics were administered to the patients using a patient-controlled analgesia (PCA) pump under the guidance of anesthesiologists both before and after MMA application. The PCA pump was generally discontinued when pain control was satisfactory, the patient desired extubation, or when postoperative nausea and vomiting (PONV) occurred.

### Outcomes and data collection

The background of the patients, including sex, age, weight, Ccr, American of anesthesiologists-physical status (ASA-PS) classification, operative procedure, and operative time were retrospectively extracted from their medical records. The primary endpoint was the LOS in the hospital. Secondary endpoints included the NRS scores on POD 1, 3, and 5, the number of patients who were administered narcotic analgesics using a PCA pump, the number of patients who discontinued PCA pump-mediated narcotic analgesic administration, and number of patients who used additional analgesics other than those pre-agreed. Notably, despite thorough collection of the NRS scores, some data were missing.

### Statistical analyses

Statistical analyses were performed using the statistical processing software EZR (version 1.61) [11]. The patient characteristics in the pre- and post-intervention groups were matched using propensity score matching to adjust for potential confounding variables. The propensity scores were calculated by logistic regression analysis and 1:1 matching with a caliper of 0.2. Sex, ASA-PS classification, age at the time of the surgical procedure, weight, Ccr, and operative time were used as matching variables. The balance between the two groups before and after propensity score matching was assessed using the standardized mean difference (SMD), with an SMD of < 0.10 indicating balanced groups. Additionally, the balance between the two groups before and after propensity score matching was compared using the Mann–Whitney U test for continuous variables, and the chi-square test or likelihood ratio test for categorical variables. After propensity score matching, the LOS and NRS scores on POD 1, 3, and 5 were compared between the groups before and after MMA application using the Mann–Whitney U test. Additionally, the chi-square test or likelihood ratio test. was used to compare the number of patients administered with narcotic analgesics using the PCA pumps, the number of patients who discontinued PCA pump-mediated narcotic analgesic administration, and the number of patients who used additional analgesics beyond those pre-agreed. All two-tailed *p*-values were considered statistically significant at *p* < 0.05.

## Results

### Patient background

**Fig. 1:**
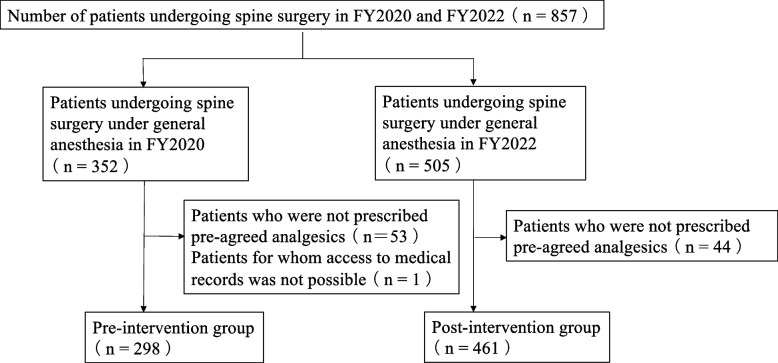
Flow diagram showing patients enrolled in the study

In total, 759 patients were included in this study, and 98 patients were excluded based on the exclusion criteria (Fig. 1). By adjusting patient characteristics through propensity score matching, the comparability between the pre- and post-intervention groups was ensured and 249 patients were included in each group (Table 1).

**Table 1:** Propensity score matching based on patient background and multimodal analgesia introduction including tramadol

	Before propensity score matching				After propensity score matching			
	Pre- intervention group (*n* = 298)	Post-intervention group (*n* = 461)	*p*-value	SMD	Pre-intervention group (*n* = 249)	Post-intervention group (*n* = 249)	*p*-value	SMD
Sex (male)^a^	169 (56.7)	250 (54.2)	0.551^c^	0.050	143 (57.4)	144 (57.8)	1.000^c^	0.016
Age (years)^b^	67.5 [56.3‒76]	72 [64‒79]	< 0.001^d^	0.345	70 [60‒78]	69 [57‒77]	0.535^d^	0.097
Body weight (kg)^b^	62 [53‒72]	60 [52.8‒70.4]	0.195^d^	0.083	62 [53‒72]	62.1 [54‒73]	0.630^d^	0.065
Ccr (mL/min)^b^	80 [62.3‒98.9]	67 [54‒88]	< 0.001^d^	0.325	76.2 [59.5‒92]	75.3 [58.5‒99.5]	0.504^d^	0.117
ASA-PS Classification			0.005^e^	0.240			0.600^e^	0.101
ASA-PS1^a^	82 (27.5)	81 (17.6)			54 (21.7)	63 (25.3)		
ASA-PS2^a^	178 (59.7)	310 (67.2)			162 (65.1)	152 (61.0)		
ASA-PS3^a^	38 (12.8)	70 (15.2)			33 (13.3)	34 (13.7)		
Operation time(minutes)^b^	82.9 [56.5‒120.5]	61.1 [43.1‒88]	< 0.001^d^	0.406	79.1 [54‒109]	64.9 [44.1‒98.1]	0.00293^d^	0.102
Surgical technique^a^			0.122^e^	0.255			0.618^e^	0.151
Cervical spine surgery^a^	65 (21.8)	71 (15.4)			52 (20.9)	50 (20.1)		
Thoracic and lumbar spine surgery^a^	233 (78.2)	388 (84.2)			197 (79.1)	199 (79.9)		
Decompression surgery^a^	79 (26.5)	139 (30.2)			67 (26.9)	73 (29.3)		
Spinal fusion surgery^a^	50 (16.8)	82 (17.8)			43 (17.3)	41 (16.5)		
Others^a^	49 (16.4)	62 (13.4)			4 (13.7)	38 (15.3)		
Decompression and spinal fusion surgery^a^	26 (8.7)	45 (9.8)			26 (10.4)	24 (9.6)		
Decompression surgery and others^a^	14 (4.7)	38 (8.2)			14 (5.6)	13 (5.2)		
Spinal fusion surgery and others^a^	12 (4.0)	19 (4.1)			11 (4.4)	10 (4.0)		
Decompression and spinal fusion surgery and others^a^	3 (1.0)	3 (0.7)			2 (0.8)	0 (0.0)		
Cervical spine and thoracic and lumbar spine surgery^a^	0 (0.0)	2 (0.4)			0 (0.0)	0 (0.0)		

*Ccr* Creatinine clearance, *ASA-PS* American Society of Anesthesiologists-physical status, *SMD* Standardized mean difference

^a^Data are expressed n (%)

^b^Data are expressed median [interquartile range]

^c^χ^2^ test

^d^The Mann–Whitney U test

^e^likelihood ratio test

### LOS and postoperative NRS scores

**Fig. 2:**
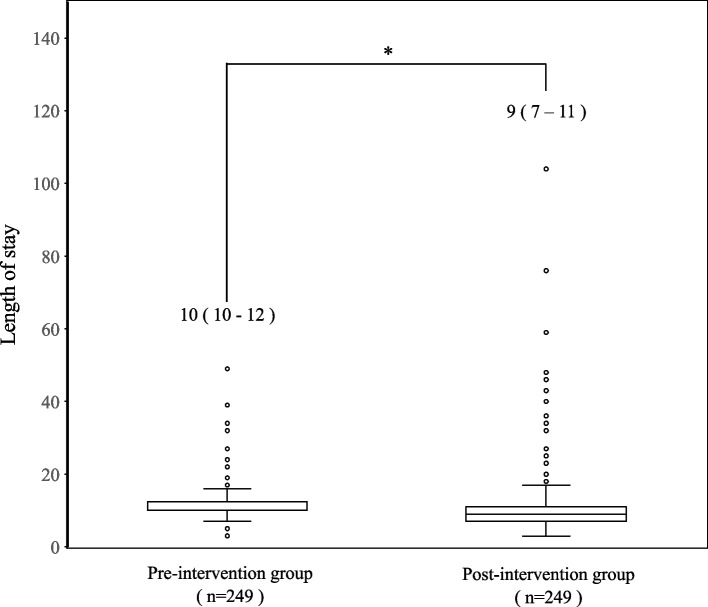
The length of stay before and after introducing multimodal analgesia (MMA) including tramadol. Values are presented as median (interquartile range). Statistical significance was determined using the Mann–Whitney U test (*p < 0.05)

**Fig. 3:**
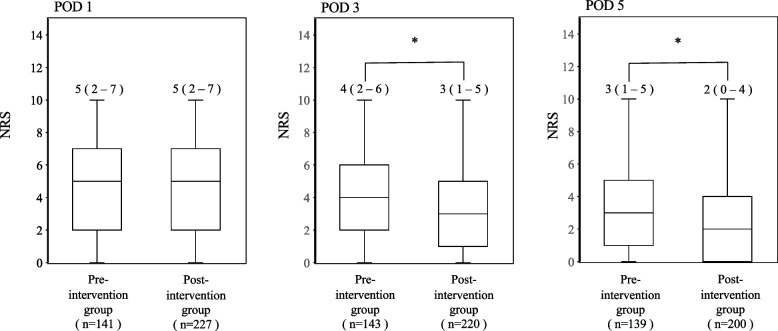
The numerical rating scale (NRS) scores before and after introducing multimodal analgesia (MMA) including tramadol. Values are presented as median (interquartile range). Statistical significance was determined using the Mann–Whitney U test (*p < 0.05). POD: Postoperative Day

The median LOS decreased significantly from 10 to 9 days after MMA application (*p* < 0.001) (Fig. 2). The median NRS scores gradually decreased following MMA application. Notably, the median NRS score on POD 1 was 5 in both groups, with no statistically significant difference. However, tramadol-including MMA application induced a significant decrease in the median NRS score on POD 3 from 4 to 3 (*p* = 0.0109), which was further reduced to 2 on POD 5 (*p* = 0.0087) (Fig. 3).

### Use of analgesics other than pre-agreed analgesics

There was no notable difference because of MMA application in the number of patients who were administered narcotic analgesics using the PCA pumps, discontinued PCA pump use midway due to PONV, or discontinued PCA pump use midway because of achieving satisfactory pain control. Notably, the number of patients who used additional analgesics beyond those that were pre-agreed decreased significantly from 38 to 6 after MMA application (*p* < 0.001) (Table 2).

**Table 2:** Use of other analgesics than those pre-agreed

	Pre-intervention group (*n* = 249)	Post-intervention group (*n* = 249)	*p*-value
Number of patients administered with narcotic analgesics using PCA pumps and number of patients who discontinued PCA pump-mediated narcotic analgesic administration			
Number of patients administered with narcotic analgesics using PCA pumps	149 (59.8)	130 (52.2)	0.104^a^
Number of patients who discontinued PCA pump-mediated narcotic analgesic administration	67 (26.9)	47 (18.9)	0.314^b^
Number of patients who discontinued narcotic analgesics due to PONV	28 (11.2)	23 (9.2)	
Number of patients discontinued narcotic analgesics because of good pain control	37 (14.9)	24 (9.6)	
Number of patients who discontinued narcotic analgesics for other reasons	2 (0.8)	0 (0.0)	
Number of patients who used additional analgesics other than the pre-agreed analgesics			
Number of patients who used additional analgesics	38 (15.3)	6 (2.4)	< 0.001^a^

*PCA* Patient-controlled analgesia, *PONV* Postoperative nausea and vomiting

Data are expressed n (%)

^a^χ^2^test

^b^likelihood ratio test

## Discussion

This study evaluated the effects of tramadol incorporation into MMA on patient outcomes in spinal surgery. Notably, the median LOS was decreased by 1 day, which was a significant difference, in patients treated with MMA. Similar to the findings of this study, Walker et al. reported a reduction of 0.7 days in the LOS because of the introduction of the MMA for spinal surgery [6]. However, the regimen of MMA employed in the study by Walker et al. differed from that of this study because of the regular administration of NSAIDs, acetaminophen, muscle relaxants, and lidocaine topical patches and administration of additional oral narcotic analgesics according to pain levels [6]. Concerns regarding persistent opioid use after surgery include misuse, abuse, addiction, and diversion [12]. The MMA regimen employed in this study may help mitigate these risks by incorporating tramadol, which is a weaker opioid, rather than a stronger one. Additionally, decreasing the LOS in the hospital can improve financial, managerial, and clinical outcomes, as it reduces the cost of patient care and minimizes the risk of nosocomial infections [13]. Although not investigated in this study, these factors likely affected the results. Further research on these factors and patient outcomes is necessary.

The median NRS scores on POD 3 and 5 after the introduction of MMA were notably reduced. This may be partially attributed to the administration of a regular dose of tramadol after dinner on POD 2. Kupers et al. reported that multiple oral doses of tramadol at the time of the surgery provided effective analgesia on the day after herniectomy [14]. Similarly, Kumar et al. reported that administering tramadol before lumbar discectomy resulted in lower pain scores and lower doses of additional analgesics [15]. Altogether, these findings support the use of tramadol for the management of acute postoperative pain.

There were no notable differences in the number of patients who used PCA pumps before and after the introduction of MMA. Additionally, similar observations were made for the number of patients who discontinued PCA pumps during the study. These results indicate that the application of MMA did not alter the quantity of narcotic analgesics administered using the PCA pumps. Notably, previous studies have reported a reduction in the use of oral narcotic analgesics [6, 7], although the quantity of narcotic analgesics used with the PCA pump has not been specified. In our study, the use of narcotic analgesics did not decrease possibly because no oral narcotic analgesics were originally used. However, the use of additional analgesics decreased after MMA application, suggesting that appropriate pain management was achieved. To the best of our knowledge, this is the first study reporting the effects of administering MMA, including tramadol after spinal surgery. Herein, the postoperative pain was managed using tramadol, rather than oral narcotic analgesics. This suggests that tramadol is a viable option for post-spinal surgery pain management.

There are several limitations to this study. First, it is a retrospective study, and we were unable to fully align patient backgrounds using propensity score matching. Therefore, caution should be exercised when interpreting the results. However, propensity score matching before or at different caliper sizes (0.1 or 0.5) also reduced the primary endpoint of hospital LOS (both, *p* < 0.001, data not shown). Second, due to the short hospital stay and the limited number of postoperative blood draws, we were unable to obtain sufficient data to assess side effects. Third, due to the retrospective design of this study, it was not possible to obtain NRS scores for every patient. Additionally, there was a lack of consistency among the healthcare providers who measured the NRS scores, which may have led to variations in the assessments. Fourth, improvements in surgical techniques may have enhanced surgical accuracy and accelerated wound healing, potentially resulting in lower postoperative NRS scores and shorter hospital stays.

Even with these limitations in mind, the results obtained from this study are very important, as they may provide valuable insights for treatment selection and approaches in the clinical setting of MMA with tramadol. Future studies should employ a prospective design, establish appropriate criteria, and ensure comprehensive data collection to enhance the reliability of findings and assess treatment efficacy and side effects with greater precision.

## Conclusion

This study evaluated the effects of tramadol-including MMA in patients who underwent spinal surgery. The results showed that this approach effectively decreased postoperative NRS scores, the use of additional analgesics, and LOS in the hospital. Altogether, these findings suggest that the introduction of MMA, including tramadol, is a beneficial approach for pain management in patients undergoing spinal surgery.

## Data Availability

The datasets generated during and/or analyzed during the current study are available from the corresponding author upon reasonable request.

## Abbreviations

MMA: Multimodal Analgesia
NRS: Numerical Rating Scale
LOS: Length of Stay
FY: Fiscal Year
ERAS: Enhanced Recovery After Surgery
NSAIDs: Non-Steroidal Anti-Inflammatory Drugs
POD: Postoperative Day
Ccr: Creatinine Clearance
PCA: Patient-Controlled Analgesia
PONV: Postoperative Nausea and Vomiting
ASA-PS: American Society of Anesthesiologists-Physical Status
SMD: Standardized Mean Difference
